# Providing safe maternity care under challenging conditions in rural Ethiopia: a qualitative study

**DOI:** 10.1186/s12913-021-06324-4

**Published:** 2021-04-09

**Authors:** Elin Mordal, Ingrid Hanssen, Andargachew Kassa Biratu, Solfrid Vatne

**Affiliations:** 1 Molde University College, Specialized University in Logistics, Faculty of Health Sciences and Sociale Care, Britvegen 2, 6410 Molde, Norway; 2 Lovisenberg Diaconal University College, Centre of clinical nursing research Lovisenberggata, 15b, 0456 Oslo, Norway; 3 College of Medicine & Health Sciences, Hawassa University, P.O.BOX 1560, Hawassa, Ethiopia

**Keywords:** Maternity care, Childbirth, Structural conditions, Qualitative method, Rural area, Ethiopia

## Abstract

**Background:**

Women’s health and the reduction in the global maternal mortality rate is a research priority worldwide. The aim of this study was to investigate the structural conditions that influence the maternity care provided for women in rural Ethiopia.

**Methods:**

A qualitative descriptive study was conducted, composed of 28 individual in-depth interviews with midwives and women who had given birth during the past 8 months, and observations of maternity care at health centres and a primary hospital. A thematic analysis was conducted.

**Results:**

The midwives do their utmost to save the lives of mothers and prioritise saving lives over providing compassionate care. Inadequate resources, such as equipment, medicine and water, affect the quality of care they provide for the birthing women. This creates a conflict between the midwives’ ideals and what conditions allow them to do. Families and the women’s network play important roles in providing care and support to the women who give birth in health facilities.

**Conclusions:**

Structural conditions make it difficult for Ethiopian midwives in rural areas to provide optimal maternity care. In addition to the availability of professional midwifery care, the expectant mothers’ families and networks also tend to provide important support and care. Further studies on how to improve the quality of maternity care from the women’s perspective are needed.

**Supplementary Information:**

The online version contains supplementary material available at 10.1186/s12913-021-06324-4.

## Background

Almost all maternal deaths occur in developing countries [1–3]. Ninety-four percent occur in low- and lower-middle-income countries. In a developing country, the risk of a woman dying from a maternal-related cause is approximately 33 times higher compared to a woman living in a developed country. In 2017, Sub-Saharan countries alone accounted for approximately two-thirds of the estimated number of global maternal deaths.

The maternal mortality rate (MMR) exposes wide gaps between rich and poor and urban and rural areas, both within a country and among countries [4]. As many women experience life-threatening complications during childbirth [5], access to essential obstetric care services, utilisation of such services, and higher quality of care are pivotal pathways to the reduction of maternal mortality and morbidity. This study is part of a larger study to investigate maternity care offered to women in rural areas of Ethiopia. The aim of this study was to investigate the structural conditions that influence the maternity care provided for women in rural Ethiopia.

If the United Nations is to attain its Sustainable Development Goals (SDGs), a continuation of the Millennium Development Goals (MDGs), by 2030, trained midwives are indispensable [6]. One of the main goals is to reduce the global MMR to fewer than 70 per 100,000 births and to have no countries with an MMR of more than twice the world average [7]. The burden of complications, injuries, and loss associated with childbirth can often be attributed to a given society’s structural conditions.

In Ethiopia, the MMR has decreased from 1250 per 100,000 live births in 1990 to 353 per 100,000 live births in 2015 [8]. According to the Ethiopia Demographic and Health Survey (2016), institutional deliveries increased from 5% in 2000 to 26% in the 2016. During the same period, a decline in home deliveries was observed, from 95% in 2000 to 73% in 2016 [9]. Ethiopia has a three-tier health care delivery system. Level one is a district or primary health care system consisting of a primary hospital (PH), health centres (HCs) and their satellite health posts (HPs). Levels two and three consist of general hospitals and specialised hospitals, respectively [10]. The government is focused on reproductive health and has achieved significant improvements in maternal health. Comprehensive national guidelines have been developed and implemented to ensure the provision of quality institutional delivery services at all levels of healthcare. Primary health care facilities constitute a cornerstone in the Ethiopian maternity care system with a special focus on the needs of the population living in rural areas [11]. Approximately 83.5% of the population live in rural areas [3], where access to health facilities is more difficult due to long geographical distances, poor infrastructure, inaccessibility, and the lack of appropriate facilities. Problems concerning access, security, and human resources are, thus, some of the factors that cause disparities in service.

Women in the highest income quantile have approximately 12 times higher skilled birth attendance than those in the lowest income quantile [12]. Many Ethiopian women have reported inadequate quality of maternity care and negative experiences in health facilities [13–15]. Poor counselling during antenatal care is deterring women from seeking skilled attendance at birth. Furthermore, many Ethiopian women do not choose to deliver in health facilities with perceived and actual poor quality of services, irregular hours, and unavailability of female care providers [16–20].

This study was conducted in Hawassa Zuriya Woreda, a rural district with more than 170,000 inhabitants, situated approximately 300 km south of Addis Ababa. This woreda [district] has one public PH and four HCs. Each of these health facilities has a catchment population of 20,000–41,000. They serve more than 12,000 women of reproductive age and assisted at 4304 births in 2017. At the time of data collection in 2019, 6772 women in the woreda were pregnant. Approximately 70% of the inhabitants have access to water from public water taps or from the river. Both the HCs and the PH have access to tap water outside the buildings. Most, but not all, of the area is supplied with mobile telephone coverage and electricity. Maternal services are free and there is an ambulance service, although it is insufficient in relation to demand. Various structural conditions influence the maternity care offered.

### Theoretical background

We found that Scott’s institutional theory is relevant for this study of structural conditions [21] to explain multidimensional phenomena in Ethiopian rural society. This institutional theory consists of three pillars: the regulatory, the normative, and the cultural cognitive pillars. While the regulatory pillar is based on a society’s legally sanctioned regulations, laws, and policies, the normative pillar includes norms, values, and roles and is morally governed. The cultural cognitive pillar is dominated by the type of social behaviours that are presumed and supported in a given culture. Scott holds that institutions are comprised of ‘regulative, normative and cultural-cognitive elements that, together with associated activities and resources, provide stability and meaning to social life’ ([21] , p. 56). He claims that organisations are more willing to change when confronted with external regulatory requirements than with normative and cultural needs.

A crucial structural condition for any institution is to have an adequate number of skilled and competent professionals. Other factors are the physical environment, such as buildings and equipment, and, in health institutions, medicines and medical technologies [22]. The organisation of facility-based treatment and care is also part of the structural condition.

The research question is: How do the structural conditions in primary healthcare in rural Ethiopia influence the maternity care provided?

## Methods

### Study design

This study has a qualitative descriptive design and features individual qualitative interviews with women who gave birth in the territorial enclave of rural Ethiopia and with their midwives. The population studied was quite homogenous.

A qualitative approach made it possible to obtain and analyse data on how a complex phenomenon is understood from the participants’ perspectives. Observation of daily life at the health facilities increased the contextual understanding and contributed to rich descriptions. This study mainly focused on structural conditions through observation and interviews with midwives.

### Participants and recruitment

A purposive sampling approach was used to identify suitable interviewees and observation participants. A preliminary sample size plan included two midwives from each of the five institutions and 25 women who had experiences with facility-based childbirth. We made these choices based on the principle described by Patton (2015), finding that the number of participants is sufficiently large and varied to illuminate the aim of the study and attain saturation during the interview process [23]. Data saturation refers to the point in the research process when no new information is discovered in data analysis, and this redundancy signals to researchers that data collection can cease. The sample size was evaluated during the data collection process.

#### Interviewees

Nine experienced midwives working at health facilities and 19 women who had given birth were interviewed. The respective facilities’ managers first identified the midwives who were working during the research period. These were then recruited face-to-face by a research assistant. Ten midwives were originally recruited, but, since three were unavailable at the time of the interviews, seven were interviewed in 2019. Two more were recruited during the 2020 data collection, bringing the total to nine individual in-depth interviews with midwives.

Potential interviewees among new mothers were randomly identified through the delivery registry book and 25 women were invited face-to-face by local health extension workers [HEWs] to participate in the project. HEWs are young women trained to identify pregnant women in their catchment area, deliver antenatal care, and connect them with the formal health system [24]. The data collection process was flexible. After 19 interviews with the women, we found that no additional data was forthcoming. Since this indicated that data saturation had been reached, we stopped the interviews at 19.

The inclusion criteria required that the midwives had to be educated at diploma or bachelor level and had worked as a midwife in a HC or PH for at least 1 year. The women interviewees had to have given birth at least once in a health facility, live in the studied area, and be able to communicate verbally with the researcher in the local language.

#### Observations

A research assistant recruited midwives for the observation part of the study. These midwives also had to be educated at bachelor or diploma level and been working at one of the healthcare facilities in question with at least 500 deliveries per annum. Eight midwives, two men and six women, working at two different health facilities were observed over a period of approximately 200 h. Birthing women at one HC and one PH were asked whether they were willing to be observed by the first author before, during, and after they had delivered their babies. All participants, whether interviewed or observed, were informed that participation was voluntary.

### Data collection

The two semi-structured interview guides developed for this study are provided in Additional file 1. Both were written in both Sidaamu Afoo, the main local language, and in English, one for the women and another for the midwives. The interview guides were pilot-tested before the interviews commenced. One question was altered to prevent misunderstandings. The pilot interviews are not part of the data presented in this study.

All the interviews were conducted by the first author. Two research assistants with master’s degrees in public health and good English and local language skills served as interpreters. All participants allowed the interviews to be audio-recorded. The interviews lasted 30–60 min and were transcribed verbatim, first in Sidaamu Afoo and then translated into English.

Except for two who preferred to be interviewed at a health facility, the interviews with the women who had given birth were conducted in their own homes. Seven of the midwives were interviewed in a separate room at their places of work and two in private homes. Seven of the midwives preferred the use of an interpreter to enable them to speak in their native language. The interviewees were invited to describe their experiences with maternity care in the health facility. Clarifications and elaborations were secured through the mirroring of statements and follow-up questions. The interviewer strove to minimise the women’s potential discomfort during the individual interviews when asking personal and intimate questions regarding their childbirth experiences [25].

The observations were conducted in 2020 in the delivery wards at the health facilities. Observation notes were written during and shortly after the observations and included information regarding the physical setting, what activities took place, who were present in the delivery ward, and interpersonal interactions. Because many women deliver at night, the observer remained at the health facility around the clock for four periods of two to 5 days each. This allowed for an in-depth view into the studied field and provided insight into the observed tasks. Intentionally or unintentionally, the observer always influences the observed [23, 26]. Therefore, a balance between participation and analytical distance is required [27]. The first author is an experienced midwife, and the wearing of a hospital uniform during observation in the delivery wards made it easier for the midwives to identify with her professionally.

### Data analysis

The analysis was thematic and hermeneutic in character, and depth of understanding was attained through a circular investigation of the interviews and literature texts [28]. The three datasets were initially analysed separately and were then seen in conjunction with each other while the researchers strived to ‘remain open to the meaning of the other person or the text’ ([28] , p. 268). Braun and Clarke’s six analytic phases for thematic analysis were used [29]. 1) The transcribed interviews and the observation notes were read several times by the researchers to familiarise themselves with the data. 2) Aspects of the data related to the aim of the study were identified and provisionally coded. 3) The codes were collated into potential themes that described the content of the interviews and observations. These themes constituted a systematic abstraction of meaning. 4) Discussions and a review of the initial themes were conducted. 5) The final themes were then defined, and their names agreed upon. During phases 4 and 5, the researchers interpreted the latent content or underlying meaning represented in the themes. 6) The first author’s preliminary text was discussed and further developed collaboratively by the authors.

Analytic credibility is obtained through presenting quotations to demonstrate the individual interviewee’s own description of thoughts and experiences. Rigour and trustworthiness were achieved through having two analysts discussing the themes. The fact that the researchers originated from different professional backgrounds, and one originated from Hawassa, Ethiopia, added value to the analysis.

## Results

The staff worked 12–24 h shifts, and they slept at the health facilities. Voluntary agreements with the employers allowed them to return home between work sessions. The midwives at the HC said that they received their basic salary every month but added that they had not been paid for weekends and holidays for the past 6 months. Therefore, they no longer worked on weekends at the HC. The only option for weekend care was at the PH. Three themes emerged from the analysis: 1) Securing the childbirth process – the midwives’ primary responsibility, 2) Lack of resources, and 3) Cooperation with the women’s social network.

### Securing the childbirth process – the midwives’ primary responsibility

The midwives regarded saving the mothers and new-borns’ lives as their main duty. They expressed a strong obligation to reduce mortality through safe services and added that they considered the preservation of the mothers and new-borns’ lives as their personal responsibility. The midwives said that they focused on health education programmes for pregnant women and did follow-up during the birthing process. They pointed out that this is what society expected from skilled birth attendants. Some of the midwives said that they believed that the community would blame them if a mother died during childbirth. One midwife summarised her responsibility in this way:

Observe the laboring mother closely, decide on medical action in time, promote health, clean beds with disinfectants, and, if necessary, immediately refer to another facility.

Even so, the women’s families and networks expressed great respect for the service provided by the midwives and shared the midwives and mothers’ focus on safe childbirth. The mothers said they needed ‘to return home healthy with the new-born in good condition’. Complications during childbirth was a major concern among both the midwives and the women.

The delivery wards were run by midwives. They managed the treatment, prescribed medications, requested laboratory analyses, and provided follow-up the birthing process. Thus, the midwives were professionally independent. The following observation illustrates this:

The woman’s contractions became weaker. The midwife decided to administer an intravenous solution, but this had little effect. An Oxytocin infusion was, therefore, administered to stimulate more powerful contractions. The birthing woman became tired, and the foetus exhibited signs of distress. Hence, a vacuum extractor was used to assist in the latter part of the expulsion. No physician was involved in either the assessments or the treatment choices.

We observed no physicians at the HC, but the PH physicians entered the birthing room when summoned by the midwife. In the event of complications such as bleeding, prolonged labour, and foetal malpresentation, the women were often referred to a better-equipped facility. The labour process, not the mothers’ comfort and care, seemed to be the midwives’ focus. An observation of the treatment of a primipara mother who was bleeding after delivering a healthy baby exemplifies this:

The midwife cleaned the area of the vulva and perineum by inserting her entire hand into the vulva to remove blood clots. She then performed powerful and painful external massage of the uterus without observing or speaking to the woman to assess her reactions.

Observation revealed that the treatment was often uncomfortable to the women, and the midwives did not seem significantly interested in reducing the mothers’ discomfort. It was common for women to experience limited attention, care and support from the midwives related to the physical and mental discomfort that is a part of normal birth process. One primipara described the care during her stay at the delivery ward thusly: ‘I was disappointed by her [midwife] approach and her attention to treat me’.

Safety first tended to be both the mothers and the midwives’ mantra. A midwife suggested ‘searching up pregnant mothers from home, mainly to reduce maternal and child mortality by providing safe services’. One of the women explained her choice to give birth at a health facility this way: ‘because there is less risk of delivery-related complications with assistance from skilled care givers. Home delivery causes both mothers and new-borns to suffer’. Another woman who had experienced care at a primary hospital said this about her preference for where to give birth:

I prefer to give birth at health facility; but not all women do. You know the women who are more aware and who fear complications and death prefer to give birth at health facility. Others are still giving birth at their home justifying their ancestors’ experiences.

This woman talked about the importance saving her life, saying she was aware that possible serious complications could be uncovered and treated rapidly at health facility or that, if necessary, the professionals could transfer her to another hospital by ambulance. Another woman said:

My plan was to get birth at health center because there are seldom health complications occur when birthing women are assisted by trained professionals. There might even be death when women are delivered at home.

One woman summarized the reasons for giving birth at a health facility this way: ‘To get lifesaving service.’

#### Systematic teaching of the benefits of using the health facilities

The midwives told the interviewer that they organised meetings where they systematically educated women in the local community regarding the benefits of professional maternity care and delivering at a health facility, thereby avoiding home delivery. The midwives also focused on the fathers with this health education, as it was important that they also understood the health message. They found it less important to inform the older generation, as the decision about where to give birth was often made by the expectant mother and her husband. The midwives’ educational outreach often changed women’s previous choices: ‘After receiving new knowledge, I decided first, and my husband supported my idea’. One of the midwives said that:

Providing regular health education to the mothers may improve their healthcare acceptance. Midwives and local HEWs should strengthen their relationships, [and] health centres and religious leaders should use their networks to influence the community to utilise healthcare services.

In spite of the changing attitudes, not all women receive quality maternity care. Some women had heard rumours of professionals being negligent ‘non-care’ givers. This made some hesitant to visit the health centres, as they were not sure what extra value these facilities added. A first-time mother who described her birth experience said this about the service she received in the maternity ward. ‘Previously I had heard that professionals were negligent non-care givers, but in reality, when I arrived, they were extremely patient care givers. Therefore, it is better for mothers and their new-borns to give birth at the hospital’.

Many had come to distrust home delivery after having experienced neonatal deaths and health complications. Others sought out skilled birth attendants in health facilities because many women in the local community did so. A woman said: ‘I will tell them that [the] health centre is important. At our coffee ceremony, we discuss the importance of facilitated birth’. She referred to the women’s informal meetings where they drink coffee and discuss questions concerning their lives, such as their birthing experiences. Thus, information was disseminated throughout the local community, and perceptions gradually changed.

### Lack of resources

The shortage of both medical equipment and essential medicines was clearly observed. The midwives mentioned various examples of shortages, such as stethoscopes, thermometers, and scales for weighing the babies, for example. This made it difficult to provide safe nursing care. The midwives blamed the Ethiopian pharmacy systems. A more efficient distribution of medicine and equipment is needed to ensure adequate supplies. A limited budget exacerbated the situation. Although several of the women we interviewed had received the medication they required, free of charge, the medicine shortage was serious, and, in most cases, the women did not receive the medication they required. One of the women had *‘*asked for analgesics after delivery, but they simply said, ‘We do not have drugs, so we will write a prescription, and then you can purchase it yourself. This woman said, ‘So, we bought the medicine I needed’. However, women from the poorest families could not afford to do so. Another woman shared the following observation about access to necessary medicines in the maternity ward:

Lack of medicines in the health center is our big problem, and we experience being referred to health facilities for lack of medicines. We strongly need better availability of necessary medicines.

However, the midwives demonstrated significant creativity to cope with lack of resources. For example, 20 cm of infusion tubing was utilised as a urine catheter, and a shortened injection needle served as an amnihook for an amniotomy.

Although both water and electricity were problematic, ‘water is our major problem’. Lack of water made it impossible for the women to wash while at the delivery ward. Systems for the delivery of electricity and water had been installed, but repairs to damaged systems were problematic. Although open incubators were available, there was no electricity to heat them. According to one of the health officers, they had exhausted their supplies of almost all medication at the HC 6 months previously, and the patients, therefore, had stopped coming. In addition, fewer patients used the facility after this problem was solved.

Sometimes birthing women were referred to another institution because ‘we lack gloves and suturing catgut for episiotomy services. These are unnecessary referrals’. For the women, this meant being moved further away from the family who normally cared for them during the pre- and postpartum periods. Having accepted the health centres as safe places for giving birth and preventing complications, referrals due to a lack of equipment were not received favourably by the women. The midwives were also dissatisfied with this practice: ‘it is not easy for us to refer mothers, but we refer them when it is impossible for us to manage the childbirth here’.

Ambulance transport to health facilities was free, but the women had to arrange for their own transport home. While the women said that ambulance transport was not always available, the midwives held that ‘transportation is no barrier at all because an ambulance is ready at any time’ and, ‘we have ambulances to manage all transportation’. At the time of the study, only one functional ambulance and one driver were available to cover the entire area. Other forms of transport, such as donkey carts or motorcycles, often had to be used instead, which could be uncomfortable and expensive.

When Ethiopia first started developing the country’s maternity care system, the offer of free ‘food, drink, and clothes for the new-born provided by the government’ was used to encourage women to deliver at health facilities with skilled birth attendants. However, the funds to provide these incentives have been depleted. For women who had received the free food, drink, and clothing for their new-borns on previous occasions, this was disappointing. Some ‘mothers, therefore, hesitated to come’. Previously, the women had been served porridge after giving birth, but this practice had also been discontinued. A woman said: ‘This means I only have a place to rest but was offered no food, drink, or coffee’. Many women, therefore, were concerned about how they would be fed. One of the women discovered that the food available at the health centre was distributed unfairly; being offered to some mothers while others had to go without*.*

### Cooperation with the Women’s social network

Observation revealed that the entire family was involved, as is usual in rural in African countries. We observed, especially in the first stage of the labour process and during the postpartum period, that the family stayed with the woman and supported her when the contractions became intense, offering her beverages and helping her to the toilet. They brought items that the mother and baby would need, such as a pillow and blankets for the birthing woman to cover herself during labour and something to dry and to wrap the new-born. The family also brought requisitions to the laboratory and prescriptions to the pharmacy and paid for the purchases themselves. On one occasion, a father went, without any objections, on a 90-min round trip to the nearest city to obtain a medication that the local pharmacy did not have in stock.

Most women had food and beverages brought from home during their stay. One woman described the role of the family and neighbours thusly: ‘All of them helped me without restrictions, for example by offering porridge, soft drink, and that like’. Many of the women expressed dissatisfaction with the fact that they were no longer received porridge or beverages for free during the stay in the maternity ward.

However, if the family was unable to afford this, the women were not offered sustenance from the facility during their stay. Some midwives paid to help these extremely poor women from their own pockets, but this was not the general practice.

During the pushing stage, birth, and delivery of the placenta, only the midwife was allowed to be present in the delivery room. Four beds were placed side by side in the delivery room at the primary hospital, and several women could give birth at the same time in the same room. As soon as the baby was born, it was entrusted to the family, preferably the mother’s mother-in-law or mother. From that point on, the family again assumed significant responsibility for the care of the mother and the new-born. They supported the woman and assisted in initiating breastfeeding. Several times we observed that the midwives went to bed shortly after a woman had given birth and left all of the post-birth care to the family. As a result, the quality of care varied according to the knowledge and resources of the family.

There was a strong female community in which the women cared for each other, as this example illustrates:

Two elderly women had come to visit a woman in the delivery ward. In the same room, a 15-year-old girl is giving birth on her own. She is experiencing repeated intensely painful contractions. One of the visitors pulls a chair over to this young girl and sits down by her bed. She holds her hand, wraps the blanket around her, stays with her, and talks to her.

This observation indicated that non-family visitors could also offer emotional support to birthing women and, thus, supplied the need for comfort, which the midwives had a limited capacity to provide. The visiting women talked, offered drinks, or often just sat there quietly, sometimes taking turns to sit with the birthing woman, two or three together.

## Discussion

The aim of this study was to investigate structural conditions that influence the maternity care provided to women in rural Ethiopia. The discussion highlighted three main themes: Meeting the need for safe childbirth with midwifery, deficiencies in the health facilities that influence the maternity care, and extensive family involvement.

### Meeting the need for safe childbirth with midwifery

Our findings indicate that saving lives tends to be more important to the midwives than support and care for the mothers. The midwives enjoyed a high degree of autonomy in their work, and the birthing women were highly dependent on their skills and expertise. Our study also expands upon this topic by focusing on the responsibility of midwives to ensure that the pregnant women received the assistance they needed, that childbirth was made safer, and that maternal mortality was reduced. This is in line with a series of articles concerning midwives and midwifery services in both high- and low-income settings in Lancet [30–34], where the midwives’ responsibility for securing the mothers’ health was exemplified by their focus on the labour process and their ability to refer to other health facilities when required. This is closely linked to the guidelines for the reduction in MMRs, especially in developing countries [1, 6]. In Ethiopia, the MDGs and the current SDGs strongly influence the authorities’ strategy for improving reproductive health and maternity care [7], and the reduction in maternal mortality receives considerable attention. A statement by the World Health Organisation (WHO) that claimed that 75% of maternal mortality can be reduced through the assistance from skilled birth attendants in healthcare facilities has had a major impact in many developing countries [35]. We find this strategy to correspond with Scott’s [21] ‘regulative pillar’, which, in the context of this study, include both national and international conditions. According to Scott, when attempting change in an organisation, it is often easiest to facilitate this in the regulatory pillar. This aligns with our findings. Although most birthing women had access to delivery wards with skilled birth attendants, changing the health behaviour of the women and the local community takes time. This may also be connected to the ‘normative pillar’, with links to religion and education, and to the ‘cultural pillar’, which concerns culturally acceptable behaviour. It is interesting that the impact of midwifery services on women’s health and survival is still not globally appreciated [36]. Despite educated midwives’ ability to improve the quality of maternity care and accomplish the health-related SDGs [36], creating the necessary structural conditions is still a significant challenge [37, 38], especially in rural areas in developing countries such as Ethiopia.

The perspective of the midwives in our study also supply additional details about structural insufficiencies that compromise the provision of healthcare, hinder the delivery of appropriate interventions, and is a source of great frustration to the midwives.

### Deficiencies in the health facilities influence maternity care

Several studies have demonstrated that women who deliver at an Ethiopian health facility are not satisfied with the quality of the care provided [13–16, 19, 39]. Ambulance transport was not always available to the women we interviewed, and sometimes no midwife was on duty when the women arrived at the delivery ward. This study revealed that the experiences of the availability of transport of the women and midwives were extremely different. The midwives pointed out that ambulance transport was available when they had to refer women to a higher level of care due to complications. It seems to be more difficult for the women to call for the ambulance when they require transport to the delivery ward. This may indicate that the professionals’ needs are emphasised over the women’s need for transport.

Our findings which should be interpreted in the light of limited geographical location. We found that the women were mostly satisfied with the maternity care they received, although many discovered that they were no longer provided with free food and items they had previously received. As some women and families were too poor to pay for the barest necessities, it was obvious that poverty affected the healthcare services provided to many birthing women in the Hawassa Zuriya Woreda. This has been found to be a problem in other parts of Ethiopia as well [18, 19].

The midwives interviewed at the HC closed the delivery ward every weekend, as they had not received weekend and holiday pay for 6 months. A consequence was that women had begun to prefer to deliver at the PHs. Unpredictable opening hours at delivery wards is a common problem in Ethiopia [39]. This counteracts the goal of securing access to safe and professionally assisted childbirth fairly close to home.

We found complex work tasks, lack of equipment, and limited time for individual birthing women may cause the midwives to reduce professional requirements and adapt to the prevailing circumstances. Paradoxically, the more successful the efforts to inform and educate women regarding the benefits of delivering their babies at the health facilities, the more the workloads and hours increased for the midwives, resulting in a poorer quality of service. Thus, the midwives faced rising demands to provide safe maternity care but with structural limitations. Without expressing this explicitly, our findings indicate that the midwives found it challenging to be unable to provide the maternity care they consider to be good practice due to the lack of medical equipment and other factors.

The reduction in the MMR and complications related to childbirth is the primary goal for improving conditions for women in rural Ethiopia and other developing countries [7]. Our results indicate that maternity care in rural areas can be unpredictable due to inadequate numbers of competent caregivers, economic resources, and equipment. The fact that these inadequacies may cause referrals to better-equipped facilities that would have been unnecessary under better circumstances exacerbates the problem. It is worth noting that such referrals were experienced unfavourably by the women and their families in this study and could be difficult to accept as the birthing women had been told that the local HCs are safe places to deliver. Thus, the scarcity of resources may affect the facilities’ reputations in the local community, making it an unattractive choice for the women in the area. However, Lipsky [40], points out that midwives’ daily prioritising can be understood as having to choose between different challenges and expending their energy on what they deem to be the most important: the safety of mother and child.

### Extensive family involvement

The results revealed a type of shared responsibility between midwives and the expectant mothers’ families, where the families, especially female relatives, provided most of the women’s pre- and post-birth care. Such family involvement is often the norm in traditional and small-scale rural or collectivistic societies [41], such as those found in the Hawassa Zuriya Woreda. In collectivistic traditions, the group or family is the core element where unity, collaboration, solidarity, and conformity are valued. Reciprocal dependency, help, and care are among the cultural aspects that safeguard and effectuate collective welfare [41]. The strong female community we observed also aligns with Scott’s [21] description of the cultural cognitive pillar. The pattern of the family’s involvement in the childbirth process also reflects Scott’s normative pillar, which concerns social behaviours that are assumed or taken for granted [42]. However, in light of the danger of complications occurring, especially during the first 24 h postpartum [43], it is important to consider the division of responsibilities between the professionals and the family [44]. Improving early postnatal care also needs attention, given the danger of episodes critical to the survival of both mothers and their new-born babies [3]. Even so, one must not underestimate the value of family care in situations of limited resources. Furthermore, such support may help the women bond with their babies and develop confidence and competence as mothers when they adjust to a new life of motherhood and parenting in their own cultural context [43].

However, the division of responsibility between midwives and families, although important, needs to be balanced. The midwives are the skilled and professional service providers, and the family must adapt to the professionals, ‘filling in the gaps’. This balance seems to be lacking to a great extent in the midwife-family relationships we observed in this study. There were almost no discussions between the midwives and the family regarding the women’s needs and how to provide the best possible care. In Hawassa Zuriya Woreda, and perhaps in other traditional rural societies as well, it is problematic that the care a birthing woman receives often depends on the resources of her social network to a great extent. This indicates that facility-based post-natal follow-up counselling is required.

### Strengths and limitations

One of this study’s strengths is the use of both interviews and observations, which resulted in richness of data. Conducting the interviews before the observations provided the opportunity for reflection regarding what to look for in clinical practice. Having a co-researcher from the Hawassa University was extremely important since data collection in a foreign country entails many challenges.

Our excellent interpreters were fluent in both languages and ensured exact and culturally appropriate translations as far as possible. Even so, meaning may be lost. The interpreters were to bridge the gap between the two languages as well as the cultures, and the risk of misunderstanding is present during both the transcription into the local language and during the translation and transcription into English. Additionally, we used an independent person to conduct a quality check of the translated transcripts. However, the collaboration with our interpreters was excellent, and linguistic problems were solved through discussion.

The presence of a researcher from abroad may have influenced the climate during the interviews as well as the responses from the participants. Additionally, the observation period provided valuable insights into the fact that limited knowledge of the context and language may affect the data collection process, the results, and the conclusions of the study.

During the analysis, we discussed our understanding in light of the cultural context to secure the claim of validity and reliability of the results. Throughout the interviews, the knowledge of the field made it easier for the interviewer to ask the participants to discuss their experiences with maternity care.

Limitations of the study include the number of participants and the fact that data was collected in a narrow territorial area. Additionally, the researcher who conducted the interviews had only slight experience regarding how to determine when saturation was achieved, leading us to interview four additional women more after declaring that saturation had been reached.

Although our study is limited to only one district in Ethiopia, and the sample size is small, the fact that the findings are supported by international research indicates that they might be transferable to similar rural districts in Ethiopia and to other rural African societal contexts. Therefore, our findings add to the knowledge base in this field.

## Conclusions

The midwives’ main duty is to provide expert care to birthing women, and the extent of a health facility’s equipment impacted on the treatment and care they were able to provide in the local health facilities. To preserve birthing women’s lives, structural conditions need to be improved to ensure access to professional caregivers and the minimum of necessary equipment and medication. The reputation of the delivery wards may be challenged when the quality of care is unpredictable. In addition to the professional midwifery care that was provided, an expectant mother’s family and network tended to provide important support and care.

It is important to understand the barriers that may hinder women’s utilisation of existing healthcare services. As many women still risk critical complications, or even death, when giving birth at home, it is important to learn more about the structural conditions for providing maternity care that need to be improved or changed. Therefore, we need more research regarding women’s own experience of delivering in rural Ethiopian health facilities. Such knowledge is important to a further increase the utilisation of professional maternity care and, thus, save women’s lives.

## Supplementary Information

**Additional file 1.**

## Data Availability

The datasets and observation notes used during this study are available from the corresponding author on reasonable request.

## Abbreviations

HC: Health Centre
HP: Health Post
HEW: Health Extension Workers
MDG: Millennium Development Goals
MMR: Maternal Mortality Rate
PH: Primary Hospital
SDG: Sustainable Development Goals
WHO: World Health Organization
