# Supplementary material for: Providing safe maternity care under challenging conditions in rural Ethiopia: a qualitative study
Source: BMC Health Serv Res. 2021 Apr 9;21:323. doi: 10.1186/s12913-021-06324-4 (PMC8033678; doi:10.1186/s12913-021-06324-4)
Supplement: Supplementary file 1 — Additional file 1. [file 12913_2021_6324_MOESM1_ESM.docx]

**INTERVIEW GUIDE** - interviews with women

Information about the interview (before the recording begins)

- Aim
- Anonymity
- Frames
- Audio recording

Are there any questions relating to the interview?

Where do you live?
How old are you?

How far is it to the Health Center/Primary Hospital from your home?
Who was planning the birth together with you?
How many children have you given birth to?

Have you gone to school?

Have the father of your baby gone to school?

How has the last childbirth been?
Who helped you during the labor proses?

Describe how you experienced being taken care of during the childbirth and the hours afterwards.

What do you need assistance to during childbirth?

Are women concerned about birth?

Did you considered to give birth somewhere else**?**

Did you celebrate the newborn when you arrived home?

Where would you recommend others to give birth?

Is there anything you would like to add, things you think are important to include in connection with this topic?

Thank you that you would attend. I really appreciate it!
